# An Individual-Based Model of the Evolution of Pesticide Resistance in Heterogeneous Environments: Control of *Meligethes aeneus* Population in Oilseed Rape Crops

**DOI:** 10.1371/journal.pone.0115631

**Published:** 2014-12-22

**Authors:** Pierre Stratonovitch, Jan Elias, Ian Denholm, Russell Slater, Mikhail A. Semenov

**Affiliations:** 1 Computational and Systems Biology Department, Rothamsted Research, Harpenden, Herts, United Kingdom; 2 Syngenta Crop Protection AG, Research Biology, Werk Stein, Schaffhauserstrasse, Stein, Switzerland; 3 Human and Environmental Sciences Department, University of Hertfordshire, Hatfield, Herts, United Kingdom; Federal University of Viçosa, Brazil

## Abstract

Preventing a pest population from damaging an agricultural crop and, at the same time, preventing the development of pesticide resistance is a major challenge in crop protection. Understanding how farming practices and environmental factors interact with pest characteristics to influence the spread of resistance is a difficult and complex task. It is extremely challenging to investigate such interactions experimentally at realistic spatial and temporal scales. Mathematical modelling and computer simulation have, therefore, been used to analyse resistance evolution and to evaluate potential resistance management tactics. Of the many modelling approaches available, individual-based modelling of a pest population offers most flexibility to include and analyse numerous factors and their interactions. Here, a pollen beetle (*Meligethes aeneus*) population was modelled as an aggregate of individual insects inhabiting a spatially heterogeneous landscape. The development of the pest and host crop (oilseed rape) was driven by climatic variables. The agricultural land of the landscape was managed by farmers applying a specific rotation and crop protection strategy. The evolution of a single resistance allele to the pyrethroid lambda cyhalothrin was analysed for different combinations of crop management practices and for a recessive, intermediate and dominant resistance allele. While the spread of a recessive resistance allele was severely constrained, intermediate or dominant resistance alleles showed a similar response to the management regime imposed. Calendar treatments applied irrespective of pest density accelerated the development of resistance compared to ones applied in response to prescribed pest density thresholds. A greater proportion of spring-sown oilseed rape was also found to increase the speed of resistance as it increased the period of insecticide exposure. Our study demonstrates the flexibility and power of an individual-based model to simulate how farming practices affect pest population dynamics, and the consequent impact of different control strategies on the risk and speed of resistance development.

## Introduction

The introduction of insecticides as a method of crop protection over the last 60 years has resulted in the development of many resistance cases amongst pest insects to several classes of active ingredient [1]. Resistance management aims to minimise the risk of resistance evolving, and has become a key objective for the crop protection and farming industries. However, the interacting effects of factors underlying the evolution of insecticide resistance in the field are not fully understood, which impedes the development of new efficient resistance management approaches. What is known is that these interactions are complex, and that it is extremely challenging to investigate them experimentally across realistic spatial and temporal scales. Mathematical and simulation models are well suited to this purpose, capturing the appropriate complexity of resistance systems to produce verifiable predictions for the evolution of resistance and insights for how this problem might be managed [2].

Alleles that confer resistance to an insecticide arise spontaneously through mutation and may already be present prior to insecticide exposure [3]. The subsequent spread of these alleles through a population exposed to insecticide is known to be determined by a combination of the biology of the pest and the prevailing environmental conditions, including the conditions of insecticide usage, e.g. timing, rate, intensity and duration of effect [4]–[6]. What are not well understood is how pest biology and the environment interact in the field and whether the environment might be manipulated to manage the spread of resistance.

Biologically, the spread of a resistance allele will depend on the relative fitness of resistance phenotypes within the mating system, genetic structure, age distribution, individual behaviour, and abundance of the population under selection. Stochasticity, particularly that of the individual probability of mating and mortality, and that occurring predominantly at very low population sizes and allele frequencies, will affect the overall likelihood of invasion of the allele into the population [2].

The environment in which pests reside provides the selective landscape across which individual fitness is modified. Under conditions of uniform and prolonged insecticide application, the resistance allele might be expected to be strongly selected for, and could potentially replace all susceptible alleles. Where the insecticide application is heterogeneous, however, being made up of a natural or human-imposed mosaic of sprayed and unsprayed patches, the refuges afforded by unsprayed patches can allow susceptible alleles to persist [7]. Indeed, under conditions appropriate in terms of the size of refuges and their arrangement in the landscape, refuges might also arrest the spread of resistant genotypes. The activity of natural enemies, working across this mosaic of patches, might also contribute to pest resistance management by selectively removing resistant genotypes [8].

Previous approaches to modelling insecticide resistance have used deterministic techniques, such as differential equation models, to investigate the effects of particular factors or behaviours on pest ecology and genetics [6], [9], [10]. Although analytical modelling has proved successful at analysing the effects of specific interactions on population dynamics, it quickly becomes mathematically intractable where it is necessary to investigate the effects of many factors simultaneously [11], [12]. To combine and analyse suites of individual properties, which depend on genetics, age, population density and spatial location, alternative quantitative approaches are required. With the advent of powerful computers, simulation approaches that explicitly include the behaviour and properties of each individual within a population have become viable alternatives. The individual-based model (IBM) approach emphasises the importance of the individual and stochasticity, and has shown that the distinctive characteristics of a particular system may originate directly from individual behaviour [13]. It is thus essential to understand this behaviour in order to predict the dynamics of the system [14]. This paradigm shift is well suited to answer the recent calls for predictive systems ecology going beyond reductionist modelling approaches that have dominated the field [15], [16]. The growing interests in predicting the evolution of ecological systems, that are complex and influenced by individual behaviour, have made IBM an increasingly popular approach. The variety of models developed demonstrates the power and flexibility of IBM. For instance, IBMs have recently been used to analyse the spatio-temporal spread of pest insects in forests [17]–[19] and agricultural landscapes [20]–[22]. IBMs have also been applied to study the relation between movements and pesticide exposure of mammals [23], [24] as well as to study pesticide resistance management strategies, e.g. the efficacy of fumigation tactics to control pest insects in stored grain [25].

In this paper, we describe a spatially explicit IBM that includes the important biological and environmental factors which affect the evolution of insecticide resistance, and which can be tailored to specific resistance problems by adopting appropriate parameter values. We apply this model to investigate resistance development to pyrethroid insecticides in pollen beetles, *Meligethes aeneus,* infesting oilseed rape (*Brassica napus L.*) (OSR) crops in the UK. The parameterised model was used to study the importance of environmental (crop rotations) and pest management (treatment thresholds) factors on the development of resistance in pollen beetle populations.

## Pollen Beetle Biology and Resistance

*M. aeneus* is one of the most damaging insect pests of OSR [26]. In the UK it attacks the crop in spring and early summer, and is the major target of spring-applied pesticides. Adults emerge from overwintering sites in March-April, feed on pollen from a range of plant families, and then migrate to winter-sown OSR (WOSR) crops where they mate and lay eggs in the flower buds [26], [27]. Oviposition damage by adults and feeding damage by first instar larvae within the bud results in bud abscission and loss of yield. Backward WOSR and crops sown in spring (SOSR) are most at risk as the growth stages most susceptible to damage by *M. aeneus* occur after beetles have emerged from overwintering and are seeking oviposition sites. Females lay up to 200 eggs during the reproduction period which may last for as long as 2 months [26], [28]. Eggs develop to adults in approximately 30 to 55 days [28]. From late June, the new generation of adults feeds on pollen from open flowers before moving to overwintering sites without mating [26], [27].

Until recently, control of *M. aeneus* in northern Europe relied almost exclusively on pyrethroid insecticides. During the spring in the UK, beetles are often exposed to at least 2 insecticide sprays, 1 applied at the green bud stage and specifically targeting *M. aeneus*, and the other applied during flowering, targeted primarily at a coexisting pest, the seed weevil *Ceutorhynchus assimilis* [29]. The pest density thresholds developed as triggers for spraying vary considerably across Europe. In the UK it is recommended that action be taken when beetle numbers exceed 15 per plant at green bud stage for a standard WOSR crop, 5 per plants for a backward WOSR crop (e.g. one that has encountered frost damage), and 5 per plant for a SOSR crop. In many other countries, spray thresholds are lower (around 5 per plant for OSR generally), partly as a consequence of a greater proportion of SOSR crops. In reality, however, many growers are reluctant to scout for pest numbers and often spray at lower population densities, a practice encouraged by the very low cost of pyrethroids when these insecticides were still an effective control option.

Pyrethroid resistance was first reported in 1999 [30] and has since become widespread across northern Europe [31]. Progressive increases in the frequency and geographical extent of resistance have been tracked by several laboratories using a standardised bioassay methodology, yielding one of the most comprehensive resistance monitoring datasets available [31]. In the UK, resistance was slower to appear, being first documented in 2005, but has since spread to all of the major OSR-growing regions in the country [32]. Evolution of resistance has been accompanied by a progressive decline in control efficacy with pyrethroids, prompting the rapid registration of alternative classes of insecticides to which no resistance has been reported to date. There is consequently much interest in exploring factors that contributed to the appearance and spread of pyrethroid resistance in *M. aeneus* and in identifying how best to minimise the risk of resistance to newer chemicals.

## Model Description

The individual-based model of pest resistance simulates a spatially heterogeneous agricultural landscape consisting of farmers' fields and semi-natural habitats. Farmers manage their fields following a defined sequence of crop rotation. Insects invade and move around this landscape according to their life cycle, host plant preferences and dispersal abilities. Individual insects are born, develop, mate, reproduce and die according to pre-set stochastic rules. Farmers control pests by applying insecticide according to calendar dates (‘calendar treatment’) or when pest density exceeds a threshold (‘threshold treatment’). Insects with different genotypes are not equally susceptible to the chemicals applied. The selection pressure for resistant genotypes emerges from a combination of landscape features, control decisions and parameters defining the ecology of the pest and the properties of resistance genes. The model progresses on a daily time step and simulations are run over several years with changes in allele frequency tracked over this period. By varying conditions and repeating simulations, it is possible to investigate which factors or combinations of factors have most influence on the risk of resistance development. Potential integrated resistance management (IRM) strategies can be evaluated and compared *in silico*.

### Landscape structure and cropping patterns

**Figure 1:**
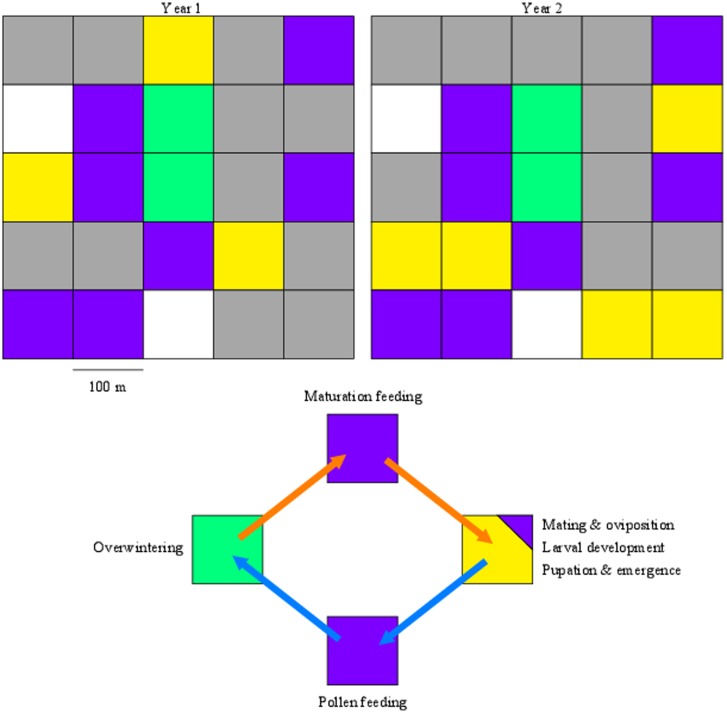
Schematic representation of a 5×5 cell landscape for 2 successive years (top) and life cycle of *M. aeneus* (bottom). Positions of cells (arable fields in yellow (OSR) or grey (non-OSR crops such as cereals), overwintering sites in green, refuges in purple, empty cells in white) are fixed at the beginning of the simulation. During the rotation cycle, the location of cells with OSR (yellow) changes as OSR is sown only once every 4 years in a field. In spring, after hibernation, adults move into refuges for maturation feeding and later colonise OSR crops for mating and oviposition (orange arrows). The next generation pupates and emerges from the soil in summer, feeds in refuges and finally moves to the overwintering sites at the end of summer (blue arrows).

The simulated landscape represents a group of spatially-heterogeneous habitats divided into a grid of square cells. A cell is the smallest spatial unit and represents an area of 1 hectare; insect position is not tracked within a cell. Considering the size of the landscape (100 cells), the same climatic and environmental conditions (e.g. minimum and maximum daily temperatures, day length) are used in all cells. A proportion of these cells are set to be uncropped habitats (e.g. woodland) that serve as overwintering sites for *M. aeneus* (Fig. 1). The rest of the landscape is divided between agricultural crops that do (e.g. OSR) and don’t (e.g. cereals) serve as host plants for *M. aeneus.* It is also necessary to include non-crop refuge cells where the pest feeds on pollen before and after hibernation, which are safe from insecticide exposure. The host range of *M. aeneus* has been limited in the simulations to 2 OSR crops, winter and spring, and wild plants attractive to *M. aeneus* growing in non-crop refuges. OSR crops are grown in every field (cells allocated to a farmer) in a strict 4 year rotation. The type of OSR crop is randomly selected between SOSR and WOSR with probabilities of 5% and 95%, respectively, representing cropping practices in the UK [33]. The sowing date is set randomly within a week of 1st April for SOSR and of 1st September for WOSR. Phenological models for WOSR and SOSR were based on published work [34]. The SOSR model has a shorter period between emergence to onset of flowering, which is determined only by thermal time and photoperiod without vernalisation. In non-crop refuges, wild plants provide pollen and oviposition sites through the year. The density of plant was kept constant for OSR and wild host at 180 plants/m^2^ and 150 plants/m^2^, respectively.

### Population dynamics of *M. aeneus*

At model initialisation, an adult population is added to the overwintering sites of the landscape. In spring, when the mean air temperature exceeds 9°C consecutively for 5 days, insects move from their overwintering sites to suitable host plants (OSR or non-crop plants). These adults feed for a period of 400 day degrees (base 0°C). Then the adults are able to reproduce during a period of 1000 day degrees (base 0°C). Non-gravid females have a mating probability dependent on the density of males present in the same cell, i.e.: 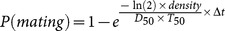
(1)


where *density* is the current adult male density in the cell, 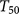 =  3 days and 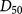 =  25 males/m^2^. This is the expected duration and male density to observe 50% of mated females, respectively. Females only mate once and the partner genotype is chosen randomly according to the distribution of male genotypes present in the cell at the time of mating. Gravid females lay 10 batches of eggs every 85 day degrees (base 0°C). Each time, the number of eggs laid is drawn from a uniform distribution of minimum 15 and maximum 35, i.e. females lay 250 eggs on average [28]. The genotype of each egg is assigned randomly from the parent's offspring genotype table. Eggs develop successively into larvae, pupae and young adults in 100, 250 and 600 day degrees (base 0°C), respectively. The next generation of adults moves to the overwintering cells in autumn when the mean air temperature falls below 12°C for 5 days.

During its life cycle, many factors (other than insecticides) influence the survival of *M. aeneus*. Very wet conditions can enhance pathogen attack, and drought can limit larvae survival [35]. Rates of predation and parasitism are dependent on the location of beetles within a field and the surrounding habitats [35], [36]. *M. aeneus* has a number of natural enemies [37] and is part of a complex food web. In the model, however, the probability of natural mortality (predation, parasitism, starvation) only depends on the life stage of an individual, the host type (wild or OSR) and the density of individuals occupying the same ecological niche (larvae or adults). The overall mortality probability is calculated at each time step as:
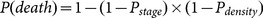
(2)


where 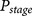 is the expected mortality from predation and parasitism and 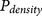 the density-related mortality. 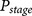 is calculated according to the time elapsed during the simulation step, 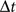:
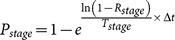
(3)


where 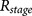 is the expected mortality rate (%) over the stage duration 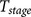 (day degrees, base 0°C) and 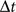 the accumulated day degrees (base 0°C) during the simulation step. Density-related mortality only occurs if the current density of competing individuals in a cell exceeds a threshold 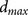, i.e.:
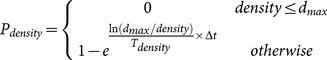
(4)


where 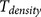 (days) is the time required for the population to decline to 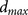 and 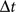 the number of days elapsed during a single simulation step, i.e. 1.

Egg and pupa stages are not subject to density-related mortality. 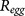, 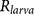 and 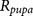, i.e. expected mortality rate over egg, larva and pupa stages, were all set to 33% and considered to be independent. So, from birth to the end of the pupa stage, the mortality from predation and parasitism is expected to be 70%, i.e. 1-(1−0.33)^3^. Larval density mortality parameter 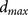 was set to 100 and 62 insects/plant for OSR host and wild host, respectively, to reflect the higher suitability of OSR crops to the pest. 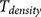 was set to 5 days simulating a strong competition for resources among larvae. With these parameter values and in the absence of insecticide treatments, the average overall mortality from birth to the end of the pupa stage recorded in a continuous 300 years simulation with typical weather generated for Rothamsted was about 75%, within the range of values (66% to 96%) reported in the literature [35], [38]. There is a lack of information from the literature on the mortality of *M. aeneus* after pupation. Consequently, from pupation, the population was controlled with 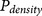 rather than 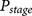. Density-related competition among adults was set to occur in 2 phases, before and after overwintering. Before winter, adult density mortality parameter 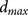 was equal to the values for larvae in OSR and wild host. After winter, and for the rest of the season, 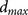 was decreased to 70 and 44 insects/plant for OSR host and wild host, respectively. To decrease the intensity of the competition for adults, 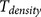 was set to 100 and 50 days before and after hibernation, respectively.

Before and after hibernation, dispersal characteristics of *M. aeneus* adults are determined by its life cycle and host preference. The frequency of movement is related to local environmental conditions e.g. host species, plant stage, and insect density. If an insect resides in a cell without any host plant, the insect is forced to move, i.e. has a probability of movement set to 1. Otherwise, this probability will depend upon the host species (OSR or wild) and the density of adults *d* within the cell, i.e.:
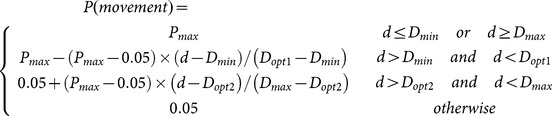
(5)


where 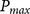 is the maximum daily movement probability, set at 0.35 and 0.65 for OSR and wild host, respectively. The probability decreases to a minimum of 0.05 as the density of insects increases from 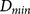 to 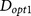. The probability then remains constant at this minimum until the density exceeds 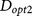. At this point, the probability increases linearly to 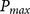 when the density increases to 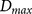. For OSR, the threshold densities 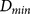, 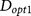, 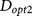 and 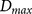 were set to 2.5, 10, 70 and 100 insects/plant, respectively. For wild hosts, the threshold densities 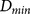, 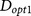, 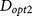 and 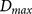 were set to 1.5, 6.2, 44 and 62 insects/plant, respectively. The higher probability of movement at low and high densities forces the adults to move to areas where enough potential mates are found but avoids overcrowding.

Although *M. aeneus* adults are known to travel upwind to attractive hosts [39], a simple model relating movements to distance and cell attractiveness (e.g. host preference) and not accounting for any wind effects was chosen. Once an insect is set to move within the grid, a distribution of potential destinations is constructed according to the distance and attractiveness of neighbouring cells:
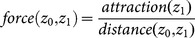
(6)


where 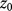 is the currently occupied cell, and 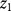 a potential destination. The 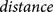 function is the shortest Euclidean distance between the centres of the cells arranged in a torus. The value of 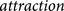 for a cell depends on the host type present, i.e. 1 and 0.2 for flowering OSR and wild host, respectively. The probability for a cell to be selected as destination is proportional to its 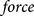: 
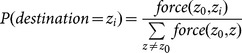
(7)


The movement is instantaneous and occurs at the end of the simulation step. In spring, at the end of their period of hibernation, adults are attracted to OSR and wild hosts following the rule described above. Using the same procedure, young adults are attracted to nearby overwintering sites before winter where they remain stationary until next spring. All non-adult life stages are considered immobile.

**Figure 2:**
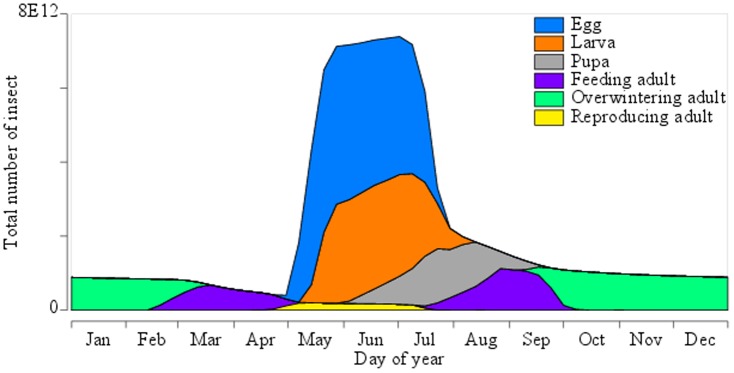
Population size and structure in the absence of insecticide treatments. The size of the population and proportions of insects at different stages of development across the entire landscape were averaged over 300 years of typical daily weather generated for Rothamsted. Green, purple and yellow colours represent overwintering adults, feeding adults and reproducing adults, respectively. Blue, orange and grey colours represent: eggs, larvae and pupae.

**Figure 3:**
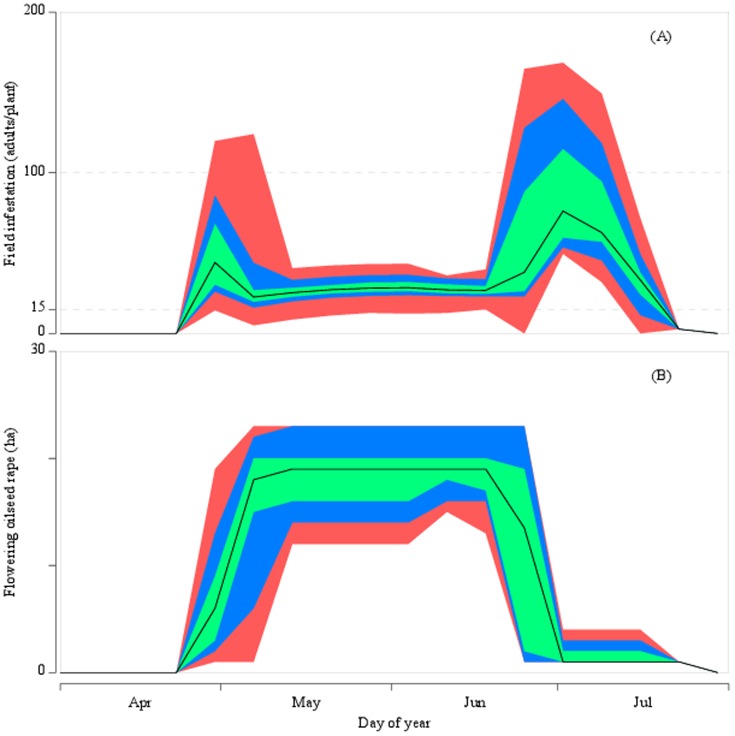
Pest pressure and oilseed rape area during the reproductive period. The weekly distributions of the average number of insects per infested field (A) and the average area of flowering oilseed rape (B) were derived from a continuous simulation of 300 years of daily weather generated for Rothamsted. In this simulation, 95% of oilseed rape crops were WOSR and fields were not controlled with insecticides. The minimum/maximum, 5^th^ and 95^th^ percentiles, 1^st^ and 3^rd^ quarter envelopes are shown in red, blue and green, respectively. The median of the distribution is shown in black.

With this parameterisation, initial runs were made to assess the outcome for population dynamics (Figs. 2 and 3). Without control, the population reaches landscape capacity and is limited by density mortality. At such numbers, the recommended treatment threshold of 15 adults/plant on WOSR for the UK is well exceeded in the majority of fields throughout the reproductive period. The density of adults in OSR crops reaches its highest levels at the beginning and end of this period. First, as more WOSR crops begin flowering, the population has more area to colonise and the density per field decreases. At the end of the reproductive period, flowering SOSR gradually disappears from the landscape and this concentrates the adults in fewer fields and increases their density.

### Incorporation of insecticide treatments and resistance

Within the model, farmers can decide to treat their crops with an insecticide based either on calendar dates (e.g. 10 days after onset of flowering) or when the insect density exceeds a prescribed threshold (e.g. 15 adults/plant). Only 2 treatments can be applied in 1 season. The model was parameterised for treatment with lambda cyhalothrin, a pyrethroid insecticide widely used against *M. aeneus* prior to the development of resistance to pyrethroids in the UK.

The probability of mortality due to insecticide treatment depends on the duration of exposure, the degradation profile of the compound and the genotype of the insect (homozygous-susceptible SS, homozygous-resistant RR or the heterozygote RS). After application, the chemical gradually loses its effectiveness due to chemical degradation, weather conditions and plant growth. At each time step 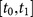, the model computes for each insect the dose it has received by integrating the treatment efficacy over the time step:
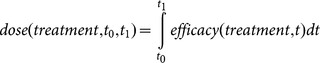
(8)


This follows Haber's rule [40]. The treatment efficacy at time 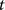 is defined as: 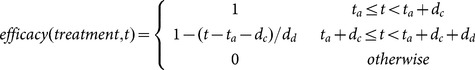
(9)


where 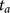 is the time of treatment application, 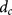 the duration of maximum efficacy 1, and 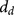
the duration of declining efficacy from 1 to 0. According to its susceptibility factor 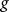, the damage from 1 treatment inflicted on an insect is defined as:
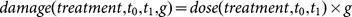
(10)


Step by step, the damage from encountered treatments accumulates and the probability of mortality increases:
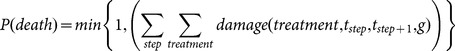
(11)


where 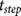 is the time at the beginning of the step. When an insect is no longer exposed to any insecticides, the damage inflicted on it is reset to 0.

To simulate the selection pressure for resistance, mortality schedules need to be defined for each of the 3 genotypes representing a monogenic resistance trait. Unfortunately, these data are not available since it is impractical to rear *M. aeneus* in the laboratory in order to obtain large numbers of insects of specific genotypes. Instead, we derived parameters defining the differential effect of the insecticide on these genotypes using data from field trials at sites with differing frequencies of pyrethroid resistance (see next section). Susceptibility factors for homozygote-susceptible and -resistant individuals are described by the parameters 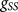 and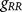, respectively. The susceptibility factor for heterozygotes 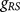 is a weighted average of the homozygotes:
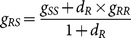
(12)


where 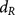
is the weight (or dominance) attributed to the R allele.

### Calibration of responses to lambda cyhalothrin using field trial data

**Figure 4:**
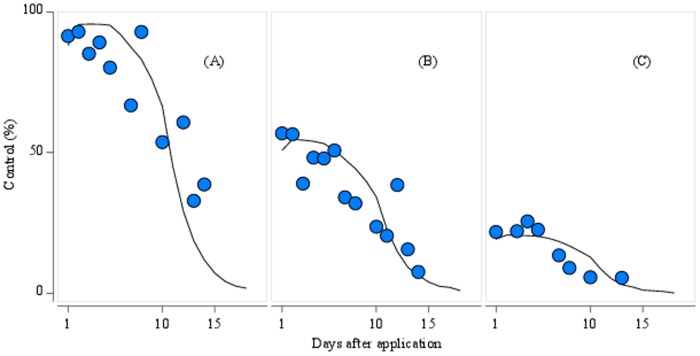
Observed and simulated lambda-cyhalothrin control for 3 levels of resistance. The measure of control is the reduction in insect number in treated plots relative to untreated plot population. Trials were pooled to reflect 3 different levels of pyrethroid resistance: (A) low resistance (75%–100% control), (B) moderate resistance (35%–75% control), and (C) high resistance (5%–35% control). Mean daily observed values averaged from field trials are shown as blue circles. Average simulated control is shown by the black line.

Data used for model calibration were combined from 48 independent trials of the efficacy of lambda cyhalothrin against *M. aeneus* carried out in 10 countries. In these trials, fields were divided into plots. Some plots remained untreated (check plots) and the others were treated with lambda cyhalothrin at field application rates. The control achieved using the pyrethroid was determined by counting insects present at regular intervals post-treatment, and calculating the reduction in insect numbers relative to check plots. Therefore, for a single trial, the insecticide control was measured at multiple time points and Fig. 4 shows the daily control averaged from all trials. The results were pooled for 3 categories of trials, considered to reflect 3 different levels of pyrethroid resistance in the locations concerned. These categories were: low resistance (75%–100% control, Fig. 4A, 23 trials), moderate resistance (35%–75% control, Fig. 4B, 16 trials), and high resistance (5%–35% control, Fig. 4C, 9 trials). The lambda cyhalothrin efficacy curve is described by 2 parameters, 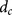 and 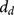
(duration of maximum and declining efficacy, respectively). The 3 genotype susceptibility factors are described by 3 parameters, 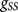, 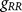 and 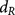. To derive these 5 model parameters, we compared experimental trial data to simulation outputs. In the virtual experiment, “fields” contained 3 replicate “plots” per treatment (check and treated plots). Initially, 150,000 insects were randomly placed across the field. The genotype of these insects was also randomly chosen from the Hardy-Weinberg distribution according to the level of resistance to *lambda cyhalothrin*. The resistance allele frequency was set to 0.5%, 25% and 55% for areas with low, moderate and high resistance, respectively. During the trials, the insects were free to move between plots, with daily probability of movement fixed at 40%. Their destination plot was selected at random without accounting for any distance effect. The model recorded daily control obtained in treated plots relative to the check plots. These control values were averaged per initial resistance frequency. These daily averages were then compared to the field data average for the corresponding initial resistance frequency using normalised Root Mean Square Error (nRMSE). This error between experimental data and model predictions was minimised during the parameter calibration process using an evolutionary algorithm [41]. The parameters were set to the median values of 5 out of 10 independent calibrations giving the lowest nRMSE, i.e. 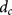 = 4.7 days, 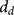 = 6 days, 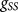 = 0.86, 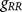 = 0.003 and 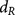 = 48.6.

## Simulation of the Impact of Cropping Patterns, Treatment Decision and Gene Dominance on the Development of Resistance

### Definition of a resistance ‘outbreak’

**Figure 5:**
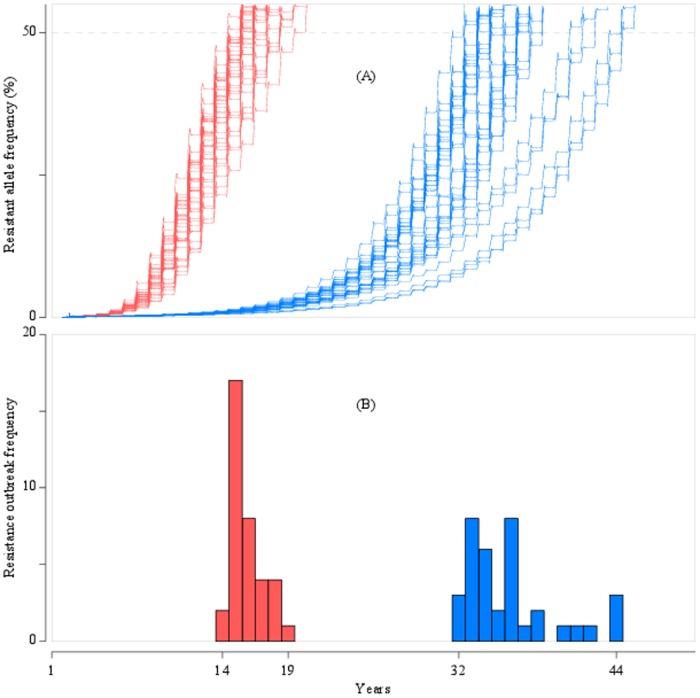
Resistance evolution and resulting distribution of time before resistance outbreak in a Monte Carlo experiment. Increase in resistance allele frequency (A) and distribution of the number of years for this frequency to exceed 50% (B) in a Monte Carlo simulation for 2 inheritance modes: dominant (red) and intermediate (blue). In this scenario, treatment decision followed the recommended high threshold (15 and 5 insects/plant for WOSR and SOSR, respectively) and 95% of oilseed rape was WOSR. The number of repetition was 36 simulations for both scenarios.

Adult individuals can be exposed through their lives to a number of insecticide treatments. The selection pressure for resistance arises from the nature of the treatments and the difference in fitness of the insects. After a treatment, susceptible individuals are killed in a greater proportion than the resistant ones, hence increasing the frequency of a resistance allele. The modelling of these interactions leading to selection encompasses 2 key stochastic processes. First, the movement of individual insects across the landscape determines the likelihood and extent of exposure to insecticide. Second, the mortality following exposure also includes stochastic elements. Therefore, the frequency of the resistance allele progresses at a different pace between repetitions of a Monte Carlo simulation experiment, as illustrated in Fig. 5.

In a single simulation, an outbreak of resistance is defined as the point when the frequency of the resistance allele exceeds 50% in the population, as in [5] but for at least 6 months because that frequency might decrease naturally after mating. For each scenario, e.g. a combination of WOSR proportion and a treatment decision, the distribution of the number of years before an outbreak of resistance was computed from 36 single simulations. These 36 repetitions were obtained by simulating the scenario for all combinations of 6 landscape arrangements and 6 sets of daily weather. The 6 different 10 ×10 cell landscapes were randomly generated with a fixed proportion of different cell types: 80 arable fields, 10 non-crop refuges, 5 overwintering habitats and 5 cells left empty. The 6 sets of 50 years of daily weather were generated using the LARS-WG weather generator as described in [42] for Rothamsted and the time period 1980–2010.

For all the simulations of the 8 scenarios described below, an initial population of 1 billion individuals was generated by placing hibernating adults randomly in an overwintering cell. The sex of an individual was randomly drawn from a Bernoulli distribution with equal probabilities. The genotype of each individual, i.e. SS, RS or RR, was randomly assigned from the Hardy-Weinberg distribution with an initial resistant allele frequency of 0.1%. The initial number of individuals was chosen below the average number of adults observed in untreated simulations to allow the population to grow during the first year of the simulation and prevent early and excessive insecticide applications in threshold scenarios. At the same time this number should be large enough for a number of heterozygotes individuals to exist at the initial resistance allele frequency.

### Cropping patterns and treatment decisions

Using this approach, we investigated the effect of cropping pattern and treatment decision on the development of resistance in a full factorial experiment. The two factors investigated were (1) the proportion of WOSR and (2) the treatment decision. For the proportion of WOSR, two levels were considered: 95% (W95), which is representative of UK farming practices, and 75% (W75). These two levels were combined with 4 control strategies: (C1) 1 calendar treatment 10 days after the onset of flowering; (C2) 2 calendar treatments 10 and 20 days after the onset of flowering; (HT) set to the recommended threshold for winter (15 insects/plant) and spring (5 insects/plant) sown OSR in the UK; and (LT), a lower treatment threshold on WOSR crops of 5 insects/plant.

### Dominance of resistance allele

**Figure 6:**
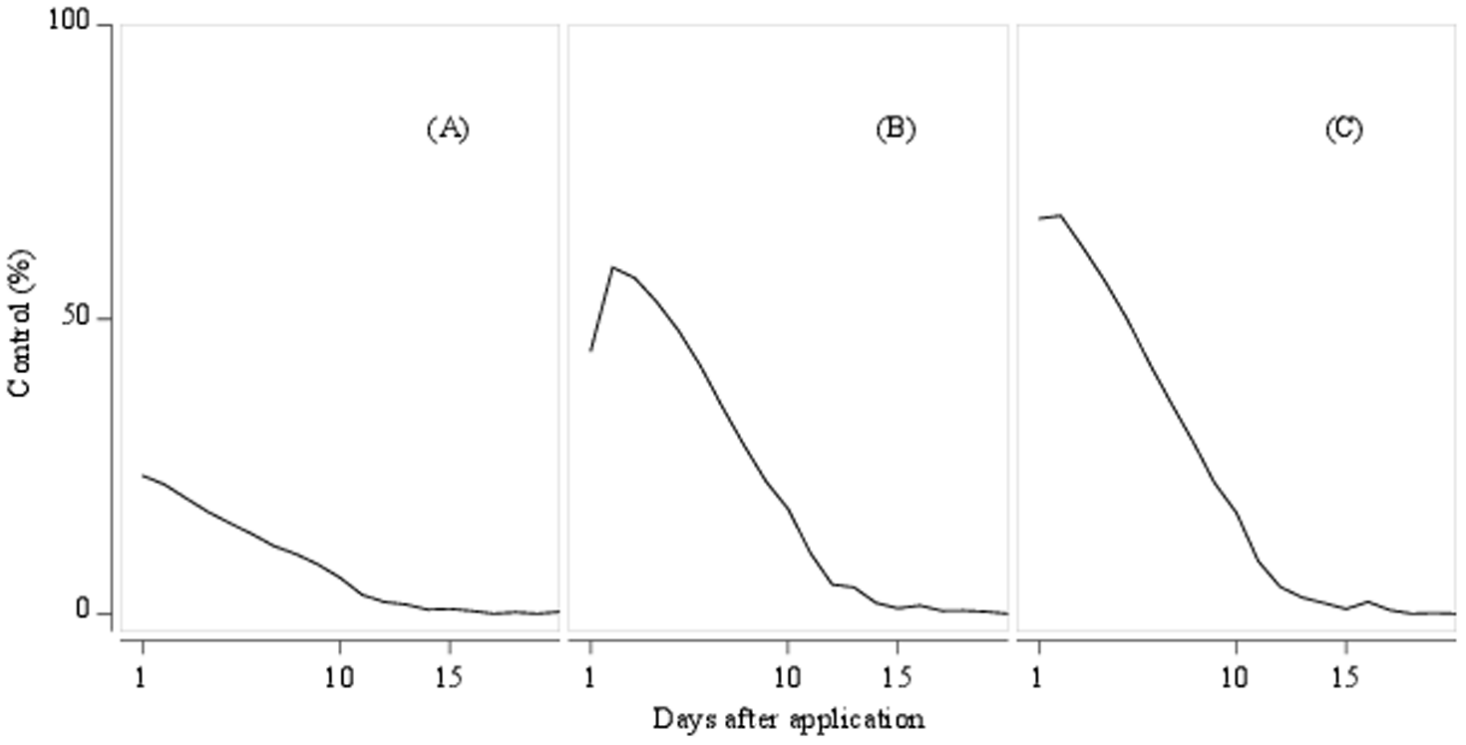
Simulated lambda-cyhalothrin control at resistant allele frequency of 50% for dominant, intermediate and recessive inheritance. The measure of control is the reduction in insect number in treated plots relative to untreated plot population. The control is simulated for 3 inheritance modes: (A) dominant, (B) intermediate and (C) recessive. For the 3 modes, the frequency of the resistant allele was set to 50%.

During calibration using field trial data, the dominance parameter 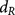 was optimised along with the other parameters. The resistant allele was found to be incompletely dominant with 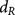 = 48.6. However, the simulations were run for 3 modes of inheritance, dominant (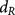 = 48.6), intermediate (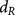 = 1) and completely recessive (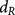 = 0) in order to compare the speed of resistance development. Other genetic parameters were kept constant. The control achieved by a single lambda-cyhalothrin treatment on a population with a resistant allele frequency of 50% is illustrated for the 3 modes of inheritances in Fig. 6.

## Results and Discussion

**Figure 7:**
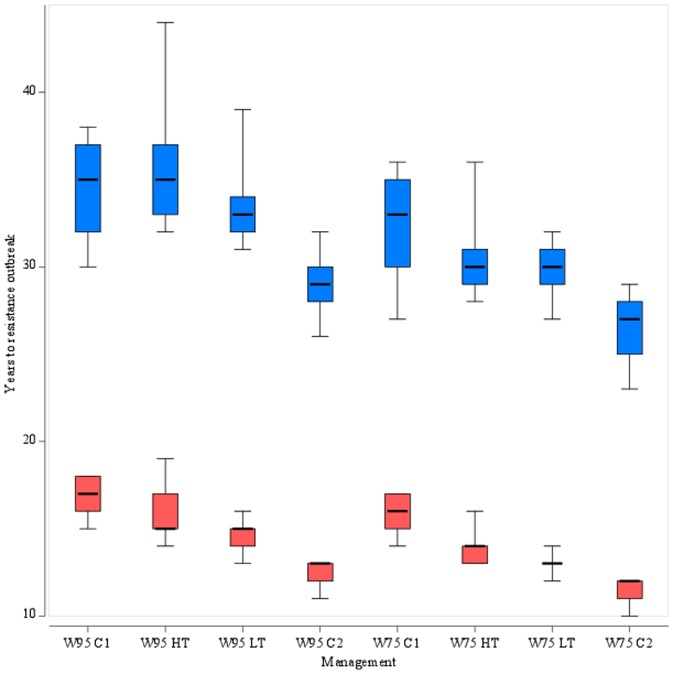
Sensitivity of projected duration before resistance outbreak to sowing practices and treatment decisions. Box plots of the distributions of the number of years for the resistant allele frequency to exceed 50% with a dominant inheritance (red boxes) and intermediate inheritance (blue boxes) for eight combinations of treatment (C1: single calendar treatment; C2: double calendar treatment; HT: high threshold treatment; LT: low threshold treatment) and sowing practices (W95: 95% WOSR; W75: 75% WOSR). Box boundaries show 25^th^ and 75^th^ percentiles, whiskers show minimum and maximum, thick horizontal line shows the median. The number of repetition was 36 simulations for each combination.

The distributions of the number of years required for a resistance outbreak for all 8 scenarios and with dominant and intermediate modes of inheritance are presented in Fig. 7. The spread of the resistance allele with recessive inheritance was very limited, with the frequency of the resistance allele failing to reach even 2% after 50 years under all 8 scenarios. This absence of resistance development with a recessive mode of inheritance is not surprising, given that at a low starting frequency most resistance alleles are present in heterozygous condition. The lack of any selective advantage for heterozygotes is a major constraint on resistance evolution and underpins strategies for resistance management under conditions that potentially allow the expression of the heterozygote phenotype to be manipulated, e.g. in the high dose/refuge strategy for crops genetically engineered to express insecticidal toxins [43]. With intermediate dominance, heterozygotes survive exposure with greater probability compared with susceptible homozygotes and as the frequency of the resistant allele increases, mating between heterozygotes becomes more frequent. This produces more resistant homozygotes with the most potent resistance phenotype. As the mode of inheritance approaches complete dominance, the speed of resistance development is maximised (Fig. 5) [44]. The difference between a dominant and intermediate mode of inheritance was consistent across all of the control scenarios investigated (Fig. 7).

In general, differences in the simulated speed of resistance development could be related to the number of treatments likely to be applied. Scenarios with a single calendar treatment (W95 C1) and high-threshold-based treatment (W95 HT) led to the slowest progression of resistance (Fig. 7). The single calendar treatment, imposed 10 days after the onset of flowering irrespective of the status of *M. aeneus*, might not always be synchronous with a high pest density because insects might leave the field earlier or colonise it later. On the contrary, the high threshold treatment ensures that a significant number of insects have colonised the field when the treatment is applied, which means that insecticide is applied later than under the low threshold (W95 LT) regime. Two applications are possible with the high threshold treatment but are less likely than with the low threshold treatment. As a consequence, resistance developed faster under the W95 LT than under the W95 HT and W95 C1 scenarios. However, the highest frequency of treatments resulting in the fastest resistance spread was achieved in the double calendar treatment scenario, W95 C2. This supports the argument that application of pesticides based on pest scouting using realistic and experimentally-validated pest thresholds contributes to minimising insecticide applications and delaying the spread of resistance [45].

Interestingly, the scenarios in which the proportion of SOSR was increased from 5% to 25% increased the speed of resistance development compared to their W95 counterparts (Fig. 7). The interactions between sowing date and the population dynamics of *M. aeneus* are likely to be complex and critically dependent on the proportion of the two crops that are cultivated. On the one hand, a large-scale move from WOSR to SOSR could greatly reduce the availability of preferred habitat after overwintering leading to suppression of pest densities, need for insecticide applications and consequently the risk of resistance. In contrast, a relatively minor change in cropping practice as simulated here prolongs the period over which favourable hosts are available, thereby increasing the likelihood of multiple insecticide treatments and the selection pressure for resistance.

While it might be tempting to relate the predictions to the historical case of *M. aeneus* resistance to pyrethroids, the apparent match between the number of years it took for pyrethroid resistance to become visible in the field (nearly 2 decades, [32]) and the number of years simulated to reach an arbitrary resistant allele frequency of 50% is accidental. Important factors that were not modelled here include long range migrations and the evolution of cropping patterns over the last decades. The model was designed to provide qualitative comparison between insecticide resistance management strategies. Therefore, the interpretation and comparison of simulation results should not be based on statistical tests of significance which could be achieved by selecting a large enough number of replicates [46].

The model behaviour was assessed in 3 sensitivity experiments evaluating the effect of the weather, surface area of refuge, and insecticide efficacy. In these experiments, the baseline conditions presented in Fig. 7 were altered. The results for all combinations of resistance inheritance (dominant or intermediate), proportion of WOSR (W95 or W75), and the 4 treatment decisions (C1, HT, LT, C2) are given in the supplementary S1 Table. The model responded similarly for both mode of inheritance and proportion of WOSR, therefore, the results, presented in Table 1, are focussed on the dominant mode of inheritance and the W95 level of WOSR.

**Table 1:** Sensitivity of projected duration before resistance outbreak to weather, surface of non-crop refuge and compound efficacy.

		Weather				Non crop refuge				Compound	
Trigger	Baseline	Broom's Barn		Berlin		−5 ha		+5 ha		Pymetrozine	
C1	16.83	16.28	(−0.55)	17.58	(+0.75)	15.61	(−1.22)	18.08	(+1.25)	18.36	(+1.53)
HT	15.83	15.69	(−0.14)	20.06	(+4.23)	18.89	(+3.06)	14.72	(−1.11)	16.64	(+0.81)
LT	14.67	14.36	(−0.31)	15.53	(+0.86)	15.69	(+1.02)	14.42	(−0.25)	15.28	(+0.61)
C2	12.39	11.97	(−0.42)	13.53	(+1.14)	11.58	(−0.81)	12.94	(+0.55)	12.75	(+0.36)

Mean number of years for the resistant allele frequency to exceed 50% with a dominant resistance inheritance, 95% of WOSR and 4 treatment decision (C1: single calendar treatment; C2: double calendar treatment; HT: high threshold treatment; LT: low threshold treatment). Values in brackets indicate relative change in years from baseline simulations presented in Fig. 7.

A spatially-explicit individual-based model of a pest population inhabiting a heterogeneous environment requires the formulation of behavioural rules applied at the individual level. Here, the characterisation of movement frequency and destination is based on a simple model and remains the largest unknown in the model parameterisation. The actual rules and parameter values were selected by observing the simulated population patterns of cropped and uncropped cell occupancy by adult beetles. In [6], the proportion of emigrating individuals and the maximum distance they travelled were shown to influence strongly the speed of resistance development. With our model, a similar conclusion will most likely be reached. Furthermore, while the model mortality rates for egg, larva and pupa stages have been derived from published materials, the rates for adults have been chosen in order to obtain around 5% of insects reaching the end of their life cycle, i.e. dying of old age, in absence of insecticide treatments. The parameters adjusted therefore included the ‘host value’ of cropped and uncropped habitats, e.g. plant density and density-related mortality thresholds. A constant ratio of 0.625 was used to define the density thresholds for wild refuges relatively to OSR crops with the rationale that OSR crops are able to support a greater density of insect as all plants are potential hosts.

In this model, exposure to insecticide treatments is also sensitive to annual weather patterns that drive the phenology of both the insects and OSR crops and as a result their synchronicity. Therefore, we conducted the simulations at two additional locations, Broom's Barn (UK) and Berlin (Germany). As for Rothamsted, the 6 sets of 50 years of daily weather for Broom's Barn and Berlin were generated using LARS-WG [42]. Different weather patterns resulted in different pest dynamic and changed the duration of exposure, which was prolonged at Broom's Barn and shortened at Berlin. As a result, a consistent change across all scenarios was observed. Resistance developed slightly faster at Broom's Barn than at Rothamsted, by an average 0.4 years. On the contrary, more continental weather simulated at Berlin delayed the development of resistance by 1.4 years. The largest differences at Berlin were found for the W95 HT scenarios where the outbreak of resistance was delayed by 4.2 years.

The proportion of individuals exposed to insecticide treatments is another critical factor in the development of resistance. The carrying capacity of the landscape, in terms of number of individuals colonising OSR crops, depends on the surface area of non-crop refuges where the individuals compete before and after hibernation. The sensitivity of the model was investigated by varying the surface area of non-crop refuge. The number of fields and woodlands were kept constant in all 6 landscapes, but the surface area of non-crop refuge was decreased to 5 ha by randomly replacing some refuge with an empty cell, or increased to 15 ha by allocating the empty cells as refuges. In this way, the area of OSR remained constant. Calendar and threshold scenarios responded differently to these changes. Relative to the baseline simulations, the change in the surface area of non-crop refuge was positively correlated with the number of years before the outbreak of resistance in calendar scenarios. For threshold scenarios, the relation was opposite, i.e. greater surface area of non-crop refuge accelerated the development of resistance relative to the baseline simulations. The greater surface area of non-crop refuge diminished the competition among insect before and after hibernation. For this reason, OSR crop were colonised in greater numbers and insecticide treatments were applied more frequently in threshold scenarios, hence the relative increase in the speed of resistance development. The effect was opposite for calendar treatments because the number of applications remained the same. In these scenarios, a larger non-crop refuge sheltered more individuals from insecticide treatments, as in [5].

A sensitivity experiment was also conducted to analyse model responses to the efficacy of the compound. The efficacy curve of pymetrozine (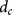 = 1.5 days, 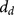 = 10.6 days) and the genetic coefficient of susceptible individuals (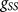 = 0.72,) were calibrated from 43 field trials following the same procedure as for lambda-cyhalothrin. Pymetrozine has a similar duration of the effect, but a shorter period at maximal efficacy. The control achieved by pymetrozine was lower than for lambda-cyhalothrin, which is reflected by a lower calibrated value for pymetrozine 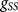. Observed and simulated controls for the pymetrozine dataset are shown in S1 Fig. Resistance to the compound pymetrozine has never been found in *M. aeneus*, therefore, a resistance allele identical to the lambda-cyhalothrin resistant allele (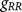 = 0.003 and 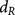 = 48.6) was created for the purpose of evaluating the impact of insecticide efficacy on the development of resistance. The control achieved by a single pymetrozine treatment on a population with a resistant allele frequency of 50% is illustrated for 3 modes of inheritances in S2 Fig. As expected, the lower susceptibility of adults to the pymetrozine compound affected all scenarios in a similar way by delaying resistance outbreaks [4].

The difference between the scenarios presented here are relatively small. For instance, the greatest difference between mean numbers of years before resistance outbreak for the scenarios presented in Fig. 7 is 5.2 years and 9.3 years for the dominant and intermediate inheritance, respectively. This narrow variation could be explained by the limited range of crop management options explored in this study. Greater differences will be expected with more complex scenarios that include insecticides with different mode of actions (used in mixture or in alternation) and a heterogeneous community of farmers applying different crop rotation and protection strategies. The models of pest and farmer behaviours would benefit from further development. For instance, the spatio-temporal mosaic of host and non-host cells generated with a simple rule ignores agronomic constraints at the farm and landscape level. Most aspects of the insect model, such as dispersal abilities and host preference were left constant and it would be interesting to study the implications of modelling these as individual and variable traits. By doing so, a more mechanistic model of intra-species competition for resources such as oviposition site and food should be considered along with an individual energy balance model [17]. Further work could also focus on improving the spatial resolution of the model. A finer scale would be required to simulate integrated pest management strategies like seed mix refuge (i.e. growers are given a mixture of traditional and modified seeds) and push-pull strategies (i.e. the growers exploit host preference and pest behaviour to their advantage [47]). Push-pull strategies are particularly relevant to the case presented here since perimeter turnip rate trap crops have been demonstrated to significantly reduce the abundance of adult *M. aeneus* in SOSR crops [29] and IBMs with appropriate spatial scale are well suited to analyse these strategies [21].

## Conclusion

We developed a spatially explicit individual-based model which can be used to simulate the evolution of resistance in a pest population in heterogeneous environments. The model incorporates important biological, environmental and management features that affect the evolution of insecticide resistance. The model can be tailored to explore specific resistance case studies by calibrating model parameters for different biological species, agricultural practices, chemical compounds or resistance management strategies. The model is stochastic in its nature and allows assessment of the risk of resistance development in response to numerous drivers. The model delivers a powerful computational tool to evaluate and compare resistance management strategies *in silico*, providing a scientific rationale for adopting the best resistance management practices.

## Supporting Information

S1 Fig **Observed and simulated pymetrozine control.** The measure of control is the reduction in insect number in treated plots relative to untreated plot population. Mean observed values from field trials are shown as blue circles. Average simulated control is shown by the black line.
(TIF)

S2 Fig **Simulated pymetrozine control at resistant allele frequency of 50% for dominant, intermediate and recessive inheritance.** The measure of control is the reduction in insect number in treated plots relative to untreated plot population. The control is simulated for 3 inheritance modes: (A) dominant, (B) intermediate and (C) recessive. For the 3 modes, the frequency of the resistant allele was set to 50%.
(TIF)

S1 Table **Sensitivity of projected duration before resistance outbreak to weather, surface area of non-crop refuge and compound efficacy.** Mean number of years for the resistant allele frequency to exceed 50% with dominant and intermediate inheritance for eight combinations of treatment (C1: single calendar treatment; C2: double calendar treatment; HT: high threshold treatment; LT: low threshold treatment) and sowing practices (W95: 95% WOSR; W75: 75% WOSR). Values in brackets indicate relative change in years from baseline simulations presented in Figure 7.
(DOCX)

## References

[1] Denholm I, Devine GJ, Williamson MS (2002) Insecticide resistance on the move. Science 297:2222–2223. 12351778 10.1126/science.1077266

[2] Bourguet D, Delmotte F, Franck P, Guillemaud T, Reboud X, et al (2013) Heterogeneity of selection and the evolution of resistance. Trends Ecol Evol 28:110–118. 23040463 10.1016/j.tree.2012.09.001

[3] Gould F, Anderson A, Jones A, Sumerford D, Heckel DG, et al (1997) Initial frequency of alleles for resistance to *Bacillus thuringiensis* toxins in field populations of Heliothis virescens. Proc Natl Acad Sci U S A 94:3519–3523. 11038613 10.1073/pnas.94.8.3519 PMC20471

[4] Georghiou GP, Taylor CE (1977) Operational Influences in the Evolution of Insecticide Resistance. J Econ Entomol 70:653–658. 915075 10.1093/jee/70.5.653

[5] Georghiou GP, Taylor CE (1977) Genetic and Biological Influences in the Evolution of Insecticide Resistance. J Econ Entomol 70:319–323. 874142 10.1093/jee/70.3.319

[6] Peck SL, Gould F, Ellner SP (1999) Spread of resistance in spatially extended regions of transgenic cotton: Implications for management of Heliothis virescens (Lepidoptera: Noctuidae). J Econ Entomol 92:1–16.

[7] Carriere Y, Ellers-Kirk C, Hartfield K, Larocque G, Degain B, et al (2012) Large-scale, spatially-explicit test of the refuge strategy for delaying insecticide resistance. Proc Natl Acad Sci U S A 109:775–780. 22215605 10.1073/pnas.1117851109 PMC3271916

[8] Schuler TH, Potting RPJ, Denholm I, Clark SJ, Clark AJ, et al (2003) Tritrophic choice experiments with Bt plants, the diamondback moth (*Plutella xylostella*) and the parasitoid *Cotesia plutellae* . Transgenic Res 12:351–361. 12779123 10.1023/a:1023342027192

[9] Jorgensen SE, Bendoricchio G (2001) Fundamentals of Ecological Modelling Oxford: Elsevier. 544 p.

[10] Tabashnik BE (1990) Modeling and Evaluation of Resistance Management Tactics. In: R. T. Roush and B. E. Tabashnik, editors. Pesticide Resistance in Arthropods. New York and London: Chapman and Hall. pp. 153–182.

[11] DeAngelis DL, Gross LJ (1992) Individual-based Models and Approaches in Ecology: Populations, Communities, and Ecosystems. New York: Chapman and Hall.

[12] Judson OP (1994) The Rise of the Individual-Based Model in Ecology. Trends Ecol Evol 9:9–14. 21236754 10.1016/0169-5347(94)90225-9

[13] Grimm V, Railsback SF (2005) Individual-based modelling and ecology. Woodstock, UK: Princenton University Press. 428 p.

[14] Grimm V, Revilla E, Berger U, Jeltsch F, Mooij WM, et al (2005) Pattern-oriented modeling of agent-based complex systems: Lessons from ecology. Science 310:987–991. 16284171 10.1126/science.1116681

[15] Evans MR, Bithell M, Cornell SJ, Dall SRX, Díaz S, et al**.** (2013) Predictive systems ecology. Proc R Soc Lond B Biol Sci 280. 10.1098/rspb.2013.1452 PMC3790477 24089332

[16] Evans MR, Norris KJ, Benton TG (2012) Predictive ecology: systems approaches. Philos Trans R Soc Lond B Biol Sci 367:163–169. 22144379 10.1098/rstb.2011.0191 PMC3223810

[17] Kautz M, Schopf R, Imron MA (2014) Individual traits as drivers of spatial dispersal and infestation patterns in a host–bark beetle system. Ecol Modell 273:264–276.

[18] Perez L, Dragicevic S (2010) Modeling mountain pine beetle infestation with an agent-based approach at two spatial scales. Environ Model Softw 25:223–236.

[19] Pérez L, Dragićević S (2011) ForestSimMPB: A swarming intelligence and agent-based modeling approach for mountain pine beetle outbreaks. Ecol Inform 6:62–72.

[20] Potting RPJ, Perry JN, Powell W (2005) Insect behavioural ecology and other factors affecting the control efficacy of agro-ecosystem diversification strategies. Ecol Modell 182:199–216.

[21] Vinatier F, Lescourret F, Duyck P-F, Tixier P (2012) From IBM to IPM: Using individual-based models to design the spatial arrangement of traps and crops in integrated pest management strategies. Agric Ecosyst Environ 146:52–59.

[22] Vinatier F, Tixier P, Le Page C, Duyck P-F, Lescourret F (2009) COSMOS, a spatially explicit model to simulate the epidemiology of Cosmopolites sordidus in banana fields. Ecol Modell 220:2244–2254.

[23] Liu C, Bednarska AJ, Sibly RM, Murfitt RC, Edwards P, et al (2014) Incorporating toxicokinetics into an individual-based model for more realistic pesticide exposure estimates: A case study of the wood mouse. Ecol Modell 280:30–39.

[24] Liu C, Sibly RM, Grimm V, Thorbek P (2013) Linking pesticide exposure and spatial dynamics: An individual-based model of wood mouse (*Apodemus sylvaticus*) populations in agricultural landscapes. Ecol Modell 248:92–102.

[25] Shi M, Collins PJ, Ridsdill-Smith J, Renton M (2012) Individual-based modelling of the efficacy of fumigation tactics to control lesser grain borer (*Rhyzopertha dominica*) in stored grain. J Stored Prod Res 51:23–32.

[26] Frearson DJT, Ferguson AW, Campbell JM, Williams IH (2005) The spatial dynamics of pollen beetles in relation to inflorescence growth stage of oilseed rape: implications for trap crop strategies. Entomol Exp Appl 116:21–29.

[27] Jonsson M, Rosdahl K, Anderson P (2007) Responses to olfactory and visual cues by over-wintered and summer generations of the pollen beetle, *Meligethes aeneus* . Physiol Entomol 32:188–193.

[28] Ekbom B, Ferdinand V (2003) Field oviposition rates and egg load dynamics of pollen beetles (*Meligethes aeneus* Fab.) (Colepotera: Nitidulidae). Agric For Entomol 5:247–252.

[29] Cook SM, Smart LE, Martin JL, Murray DA, Watts NP, et al (2006) Exploitation of host plant preferences in pest management strategies for oilseed rape (*Brassica napus*). Entomol Exp Appl 119:221–229.

[30] Hansen LM (2003) Insecticide-resistant pollen beetles (*Meligethes aeneus* F) found in Danish oilseed rape (*Brassica napus* L) fields. Pest Manag Sci 59:1057–1059. 12974359 10.1002/ps.737

[31] Slater R, Ellis S, Genay JP, Heimbach U, Huart G, et al (2011) Pyrethroid resistance monitoring in European populations of pollen beetle (*Meligethes* spp.): a coordinated approach through the Insecticide Resistance Action Committee (IRAC). Pest Manag Sci 67:633–638. 21268233 10.1002/ps.2101

[32] Nauen R, Zimmer CT, Andrews M, Slater R, Bass C, et al (2012) Target-site resistance to pyrethroids in European populations of pollen beetle, *Meligethes aeneus* F. Pestic Biochem Physiol. 103:173–180.

[33] Garthwaite DG, Thomas MR, Parrish G, Smith L, Barker I (2008) Pesticide usage survey report arable crops in Great Britain. National statistics.

[34] Habekotté B (1997) A model of the phenological development of winter oilseed rape (*Brassica napus* L.). Field Crops Res 54:127–136.

[35] Büchi R (2002) Mortality of pollen beetle (*Meligethes* spp.) larvae due to predators and parasitoids in rape fields and the effect of conservation strips. Agric Ecosyst Environ 90:255–263.

[36] Thies C, Tscharntke T (1999) Landscape structure and biological control in agroecosystems. Science 285:893–895. 10436158 10.1126/science.285.5429.893

[37] Osborne P (1960) Observations on the natural enemies of *Meligethes aeneus* (F.) and *M. viridescens* (F.) [Coleoptera: Nitidulidae]. Parasitology 50:91–110. 13854197 10.1017/s0031182000025233

[38] Buchs W, Nuss H (2000) First steps to assess the importance of epigaeic active polyphagous predators on oilseed rape insect pests with soil pupating larvae. Bulletin OILB/SROP 23:151–163.

[39] Williams IH, Frearson D, Barari H, McCartney A (2007) Migration to and dispersal from oilseed rape by the pollen beetle, *Meligethes aeneus*, in relation to wind direction. Agric For Entomol 9:279–286.

[40] Miller FJ, Schlosser PM, Janszen DB (2000) Haber's rule: a special case in a family of curves relating concentration and duration of exposure to a fixed level of response for a given endpoint. Toxicology 149:21–34. 10963858 10.1016/s0300-483x(00)00229-8

[41] Stratonovitch P, Semenov MA (2010) Calibration of a crop simulation model using an evolutionary algorithm with self-adaptation. Procedia Social and Behavioral Sciences 2:7749–7750.

[42] Semenov MA, Donatelli M, Stratonovitch P, Chatzidaki E, Baruth B (2010) ELPIS: a dataset of local-scale daily climate scenarios for Europe. Clim Res 44:3–15.

[43] Tabashnik BE, Brévault T, Carrière Y (2013) Insect resistance to Bt crops: lessons from the first billion acres. Nat Biotechnol 31:510–521. 23752438 10.1038/nbt.2597

[44] Taylor CE, Georghiou GP (1979) Suppression of insecticide resistance by alteration of gene dominance and migration. J Econ Entomol 72:105–109.

[45] Roush RT (1989) Designing resistance management programs: how can you choose? Pesticide Science 26:423–441.

[46] White JW, Rassweiler A, Samhouri JF, Stier AC, White C (2014) Ecologists should not use statistical significance tests to interpret simulation model results. Oikos 123:385–388.

[47] Cook SM, Khan ZR, Pickett JA (2006) The Use of Push-Pull Strategies in Integrated Pest Management. Annu Rev Entomol 52:375–400. 10.1146/annurev.ento.52.110405.091407 16968206

