# Supplementary figures and images for: An Individual-Based Model of the Evolution of Pesticide Resistance in Heterogeneous Environments: Control of Meligethes aeneus Population in Oilseed Rape Crops
Source: PLoS One. 2014 Dec 22;9(12):e115631. doi: 10.1371/journal.pone.0115631 (PMC4274105; doi:10.1371/journal.pone.0115631)

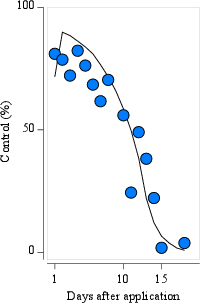

Supplement: S1 Fig — Observed and simulated pymetrozine control. The measure of control is the reduction in insect number in treated plots relative to untreated plot population. Mean observed values from field trials are shown as blue circles. Average simulated control is shown by the black line. (TIF) [file pone.0115631.s001.tif]

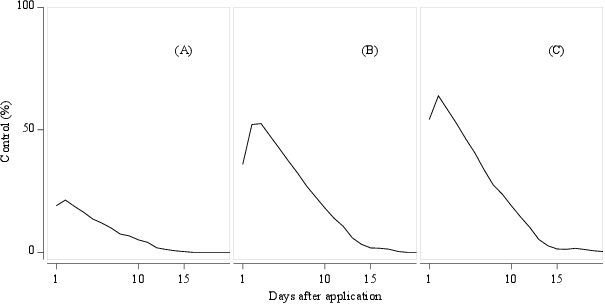

Supplement: S2 Fig — Simulated pymetrozine control at resistant allele frequency of 50% for dominant, intermediate and recessive inheritance. The measure of control is the reduction in insect number in treated plots relative to untreated plot population. The control is simulated for 3 inheritance modes: (A) dominant, (B) intermediate and (C) recessive. For the 3 modes, the frequency of the resistant allele was set to 50%. (TIF) [file pone.0115631.s002.tif]
