# Supplementary material for: An Individual-Based Model of the Evolution of Pesticide Resistance in Heterogeneous Environments: Control of Meligethes aeneus Population in Oilseed Rape Crops
Source: PLoS One. 2014 Dec 22;9(12):e115631. doi: 10.1371/journal.pone.0115631 (PMC4274105; doi:10.1371/journal.pone.0115631)
Supplement: S1 Table — Sensitivity of projected duration before resistance outbreak to weather, surface area of non-crop refuge and compound efficacy. Mean number of years for the resistant allele frequency to exceed 50% with dominant and intermediate inheritance for eight combinations of treatment (C1: single calendar treatment; C2: double calendar treatment; HT: high threshold treatment; LT: low threshold treatment) and sowing practices (W95: 95% WOSR; W75: 75% WOSR). Values in brackets indicate relative change in years from baseline simulations presented in Figure 7. (DOCX) [file pone.0115631.s003.docx]

**Table S1. Sensitivity of projected duration before resistance outbreak to weather, surface of non-crop refuge and compound efficacy.**

| **Inheritance** | **WOSR** | **Trigger** | **Baseline** | **Weather** | | | | **Non crop refuge** | | | | **Compound** | |
| --- | --- | --- | --- | --- | --- | --- | --- | --- | --- | --- | --- | --- | --- |
|  |  |  |  | **Broom’s Barn** | | **Berlin** | | **-5 ha** | | **+5 ha** | | **Pymetrozine** | |
| dominant | W95 | C1 | 16.83 | 16.28 | (-0.55) | 17.58 | (+0.75) | 15.61 | (-1.22) | 18.08 | (+1.25) | 18.36 | (+1.53) |
|  |  | HT | 15.83 | 15.69 | (-0.14) | 20.06 | (+4.23) | 18.89 | (+3.06) | 14.72 | (-1.11) | 16.64 | (+0.81) |
|  |  | LT | 14.67 | 14.36 | (-0.31) | 15.53 | (+0.86) | 15.69 | (+1.02) | 14.42 | (-0.25) | 15.28 | (+0.61) |
|  |  | C2 | 12.39 | 11.97 | (-0.42) | 13.53 | (+1.14) | 11.58 | (-0.81) | 12.94 | (+0.55) | 12.75 | (+0.36) |
|  | W75 | C1 | 15.86 | 15.11 | (-0.75) | 16.11 | (+0.25) | 14.25 | (-1.61) | 17.33 | (+1.47) | 17.03 | (+1.17) |
|  |  | HT | 13.81 | 13.44 | (-0.37) | 16.14 | (+2.33) | 14.97 | (+1.16) | 13.58 | (-0.23) | 14.36 | (+0.55) |
|  |  | LT | 13.08 | 12.78 | (-0.3) | 13.86 | (+0.78) | 13.39 | (+0.31) | 13.5 | (+0.42) | 13.56 | (+0.48) |
|  |  | C2 | 11.58 | 11.08 | (-0.5) | 12.61 | (+1.03) | 10.69 | (-0.89) | 12.36 | (+0.78) | 11.89 | (+0.31) |
| intermediate | W95 | C1 | 34.44 | 34.14 | (-0.3) | 37.14 | (+2.7) | 31.78 | (-2.66) | 37.97 | (+3.53) | 37.17 | (+2.73) |
|  |  | HT | 35.83 | 35.92 | (+0.09) | 41.54 | (+5.71) | 37.8 | (+1.97) | 32.92 | (-2.91) | 36.57 | (+0.74) |
|  |  | LT | 33.33 | 33.25 | (-0.08) | 35.25 | (+1.92) | 35.39 | (+2.06) | 33 | (-0.33) | 34.25 | (+0.92) |
|  |  | C2 | 29.23 | 29.08 | (-0.15) | 32.66 | (+3.43) | 27.65 | (-1.58) | 30.67 | (+1.44) | 29.11 | (-0.12) |
|  | W75 | C1 | 32.33 | 31.53 | (-0.8) | 33.36 | (+1.03) | 28.75 | (-3.58) | 36.53 | (+4.2) | 34.69 | (+2.36) |
|  |  | HT | 30.54 | 30.39 | (-0.15) | 37.36 | (+6.82) | 33.06 | (+2.52) | 29.72 | (-0.82) | 31 | (+0.46) |
|  |  | LT | 29.69 | 29.33 | (-0.36) | 31.5 | (+1.81) | 30.09 | (+0.4) | 31.11 | (+1.42) | 30.34 | (+0.65) |
|  |  | C2 | 26.5 | 26.44 | (-0.06) | 30.12 | (+3.62) | 25.17 | (-1.33) | 28.48 | (+1.98) | 26.42 | (-0.08) |

Mean number of years for the resistant allele frequency to exceed 50% with dominant and intermediate inheritance for eight combinations of treatment (C1: single calendar treatment; C2: double calendar treatment; HT: high threshold treatment; LT: low threshold treatment) and sowing practices (W95: 95% WOSR; W75: 75% WOSR). Values in brackets indicate relative change in years from baseline simulations presented in Figure 7.
